# Growing Season, Cultivar, and Nitrogen Supply Affect Leaf and Fruit Micronutrient Status of Field-Grown Kiwiberry Vines

**DOI:** 10.3390/plants12010138

**Published:** 2022-12-27

**Authors:** Jan Stefaniak, Barbara Łata

**Affiliations:** Section of Basic Research in Horticulture, Department of Plant Protection, Institute of Horticultural Sciences, Warsaw University of Life Sciences-SGGW (WULS-SGGW), Nowoursynowska 159, 02-776 Warsaw, Poland

**Keywords:** *Actinidia arguta*, leaf sampling, manganese, zinc, copper, boron, iron

## Abstract

The N uptake can affect kiwiberry yield and quality; however, the relationship between an increasing N dose and micronutrient accumulation in leaves and fruit is still to be elucidated. Interrelationships between essential nutrients are one of the most important issues in terms of effectiveness in plant mineral nutrition. A pattern in leaf nutrient accumulation throughout the growing period is required to indicate a suitable sampling time for the purpose of nutrient diagnostics and controlled plant feeding. The experiment was conducted on two commercially available cultivars of kiwiberry, ‘Weiki’ and ‘Geneva’, during the 2015–2016 growing seasons with an increasing soil N fertility (30–50–80 mg N kg^−1^ soil DW) to test the relationship between soil N level and leaf/fruit micronutrient concentration. The leaf Zn, Cu, Fe, and Mn concentrations significantly increased with a higher N supply in ‘Geneva’, while in ‘Weiki’ only Mn increased. Leaf B, Fe, and Mn gradually increased throughout the growing season, while Cu decreased. Between mid-July and the beginning of August, the lowest fluctuations in the micronutrient contents were recorded. The effect of the growing season on leaf micronutrient accumulation was highly significant; except for Fe, significantly higher micronutrient levels were revealed in 2016. Compared to the leaves, the growing season effect was smaller in the case of fruit micronutrient concentrations. Irrespective of cultivar, the increase in N fertilization resulted in a higher fruit Mn concentration and was insignificant in the case of other micronutrients. The results indicate that the N dose may affect the accumulation of micronutrients within a certain range depending on the tissue type and the genotype.

## 1. Introduction

Micronutrients are essential elements for all plants and are required in much smaller amounts than macronutrients [1,2]. Multiple experiments have proven the beneficial effects of micronutrients on plant growth and yield [2,3,4]. Plants show different needs for certain micronutrients, but the essential elements for all higher plants are boron (B), chloride (Cl), copper (Cu), iron (Fe), manganese (Mn), molybdenum (Mo), nickel (Ni), and zinc (Zn) [1]. Micronutrients are involved in virtually all cellular and metabolic functions, such as primary and secondary metabolism, energy metabolism, cell protection, signal transduction, gene regulation, plant defence system, hormone perception, and reproduction [1,5,6,7,8,9]. It should be stressed, however, that when concentrations of the above-mentioned ions are too high, there is the formation of reactive oxygen species, with detrimental consequences to the cells [1,6]. Moreover, micronutrients, most notably Cu, Fe, Mn, and Zn [10], are of great importance not only for plant health but also for animal and human health as key food components that are relevant for the metabolism and maintenance of tissue function [11].

Several environmental and agronomic factors can impact the availability and uptake of plant nutrients even in nutrient-rich soils, compromising fruit quality and yield [12,13,14]. The deficit in specific micronutrients, such as Fe or Mn, is a global problem [15,16]. Certain micronutrients present a challenge, partly because they have narrow dynamic ranges between the minimum requirement and toxicity level [17,18] and partly because their concentrations are subject to major fluctuations in the soil [19]. Thus, balanced plant nutrition, combined with the knowledge of nutrient interactions, is a constantly relevant issue for sustainable and healthy crop growth [20].

The task of maintaining the right supply of nutrients to plants is especially challenging and important in the case of nitrogen (N), which is in high demand due to its influence on crop yield, but it is also highly mobile [21,22,23]. Given the large amount of N fertilizers used, which impacts plant growth [23] and the pH of the rhizosphere [22], additional N supply is expected to influence the uptake of other nutrients, especially micronutrients. For example, a strong correlation between different N levels and micronutrients was discovered in the case of tobacco plants [12]. These relationships can be synergistic or antagonistic in nature [12,24,25]. Moreover, other factors including weather, soil quality traits, plant species, and variety can also affect nutrient interactions and finally their uptake [14,24,26]. In-depth knowledge of nutrient interactions and the factors accompanying these interactions is essential in order to better understand and control balanced plant feeding as well as improve nutrient use efficiency [20]. Previous research showed that an increasing N soil supply leads to a rise not only in N but also Mg and Ca concentrations in kiwiberry (*Actinidia arguta* [Siebold et Zucc.] Planch. ex Miq.) leaves, while P and K concentrations decrease [27].

In relation to micronutrients, it has previously been reported that increasing the N supply has a negative effect on Zn and B concentrations in kiwifruit (*Actinidia chinensis* var. deliciosa [A.Chev.] A. Chev.) [28]. To the best of the authors’ knowledge, there have been no other studies on this topic. This report is intended to supplement data on the relationship between an increasing soil N supply and the leaf/fruit Cu, Zn, Mn, Fe, B, and Fe statuses. The analyzed micronutrients are among those that are most frequently deficient in soils, which is usually reflected in the plant’s internal status and external appearance. In turn, based on research and field experiments carried out in Europe and the USA, the kiwiberry plant shows potential to be a future commercial success [29,30,31]. In brief, *A. arguta* is the most frost-tolerant of all commercial *Actinidia* species: the plants are resistant to biotic stresses, and the fruit are rich in health-promoting compounds and minerals [32,33]. Current practices in kiwiberry nutrition management mostly try to replicate the practices used in the cultivation of kiwifruit, which may have differing nutritional needs [30]. The first steps to set up leaf mineral sufficiency ranges for kiwiberry plants have recently been taken [34]. New information on the pattern of nutrient changes through two growing seasons in relation to shoot types in male and female plants has been described by Vance and Strik [34]. However, there are still many knowledge gaps concerning nutritional requirements and especially balanced nutrition for plants.

In light of the above, the aims of this study were: (i) to assess the fluctuation in micronutrients in *A. arguta* leaves during different growing seasons, which may help indicate the optimum time for leaf sampling for diagnostic purposes, and (ii) to show the relationship between increasing soil N levels and micronutrient accumulation in kiwiberry leaves and fruit. The two *A. arguta* cultivars, ‘Weiki’ and ‘Geneva’, were selected and tested regarding the above-mentioned issues in 2015 and 2016.

## 2. Results and Discussion

### 2.1. Leaf Micronutrient Concentration as a Function of N Level and Cultivar

In this study, changes in leaf nutrient accumulation in two kiwiberry cultivars as a result of increasing N doses were explored. In the first step, the relationship between the applied N dose and the leaf macronutrients content was established [27]. The increase in N dose reflected its increasing concentration in leaves (Table 1). It was also noted, however, that along with a better N supply, the accumulation of other macronutrients in the leaf was also changed. The increase in the leaf N, Mg, and Ca concentrations between the treatments with the lowest (30 mg N kg^−1^ DW) and the highest (80 mg N kg^−1^ DW) applied N dose, depending on the cultivar, was within the range of 17–24, 12–19, and 15–25%, respectively. In contrast, the decrease in P and K leaf content varied within the range 24–30 and 5–13%, respectively. These increases/decreases were statistically proven [27].

In the second step, the plant micronutrient nutritional status in the context of increasing N doses was assessed and the obtained results are presented in this paper. It was noted that overall soil N fertility influenced the micronutrient concentration in leaves positively, but the strength of the effect was different for each tested cultivar (Table 2 and Table 3). As described by Fageria [26], interactions between nutrients in the soil are determined not only by the level of each nutrient in the soil but also by the plant species, and sometimes differences exist between cultivars. There was a significant increase in Cu, Zn, and Fe concentrations in ‘Geneva’ leaves with increasing soil N fertility, but no effect was observed in ‘Weiki’ leaves (Table 3). An increase in Zn and Fe was noted in tomato leaves under increasing N applications of 100, 200, and 300 mg kg^−1^ [18], which is in accordance with the results for ‘Geneva’ in the present study. No effect of the application of 80 and 160 kg ha^−1^ N on Zn and Fe was observed in apple leaves during a two-year study [2]. However, only one apple cultivar was under investigation. A study of tomatoes grown under greenhouse conditions showed no interaction between increasing N fertilization and B content in leaves [18]. In the present study, different soil N levels had no effect on ‘Geneva’ leaf B concentration but did have a significant but ambiguous effect on B concentration in ‘Weiki’ leaves (Table 3). Only Mn concentrations consistently increased in the leaves of both cultivars with an increasing N supply (Table 3). A similar relationship was obtained in the case of tomato plants [18], while no effect in terms of N fertilization on Mn in an apple cv ‘Golden Delicious’ leaves was observed [2]. The accumulation of Fe, Zn, and Cu in winter wheat flag leaves was reported to be influenced by the type of N form, with the uptake of these nutrients being favorable in the case of the N-NH_4_ form [35]. The ammonium nitrate applied in this study consisted of equal amounts of the NH_4_^+^ and NO_3_^−^ forms.

### 2.2. Year Effect on Leaf Micronutrient Concentration

Weather conditions, especially temperature and precipitation, can affect the rates of plant nutrient intake and accumulation [14]. A change in soil moisture affects the soil redox potential and thus the availability of certain micronutrients such as Fe or Mn due to a change in their reduction state in less aerated soil [9]. In the present study, both growing seasons were warmer than the long-term average. Furthermore, approximately 25% more precipitation than the long-term average was recorded in 2016. Weather data accompanying the study were recently presented [36]. In the case of both analyzed cultivars, the effect of the year was highly significant irrespective of nutrient under study (Table 2). The concentrations of all examined micronutrients were significantly higher in the wetter 2016 than in 2015, with the exception of Fe, whose concentration decreased (Table 4). A similar effect regarding Fe and Mn leaf concentrations was described for apple trees but with the opposite effect in relation to Zn concentration [2]. Due to climatic changes, in an open field experiment, there is frequently a large variability in the plant chemical composition in subsequent seasons [2,27,30]. The range of variability between the years depends on the species, cultivar, or component tested. In a two-year *A. arguta* ‘Ananasnaya’ study, a two to three weeks difference in specific growth stages and vine phenology was noted between consecutive years, which was accompanied by significant variability in Fe, Cu, and Zn concentrations in leaves and no impact on B and Mn concentrations [30]. Here, compared with 2015, concentrations in ‘Weiki’ leaves in 2016 increased by 43%, 60%, 35%, and 26%, and in ‘Geneva’ by 50%, 73%, 70%, and 14% for Cu, Zn, Mn, and B, respectively, while the Fe leaf concentration decreased by 13% and 12% for ‘Weiki’ and ‘Geneva’, respectively. In a previous study on the same plants, a significant increase in every tested macronutrient concentration was observed in the leaves of both cultivars between 2015 and 2016 [27].

### 2.3. Accumulation of Leaf Micronutrient during the Growing Season: Cultivar Differences

**Figure 1:**
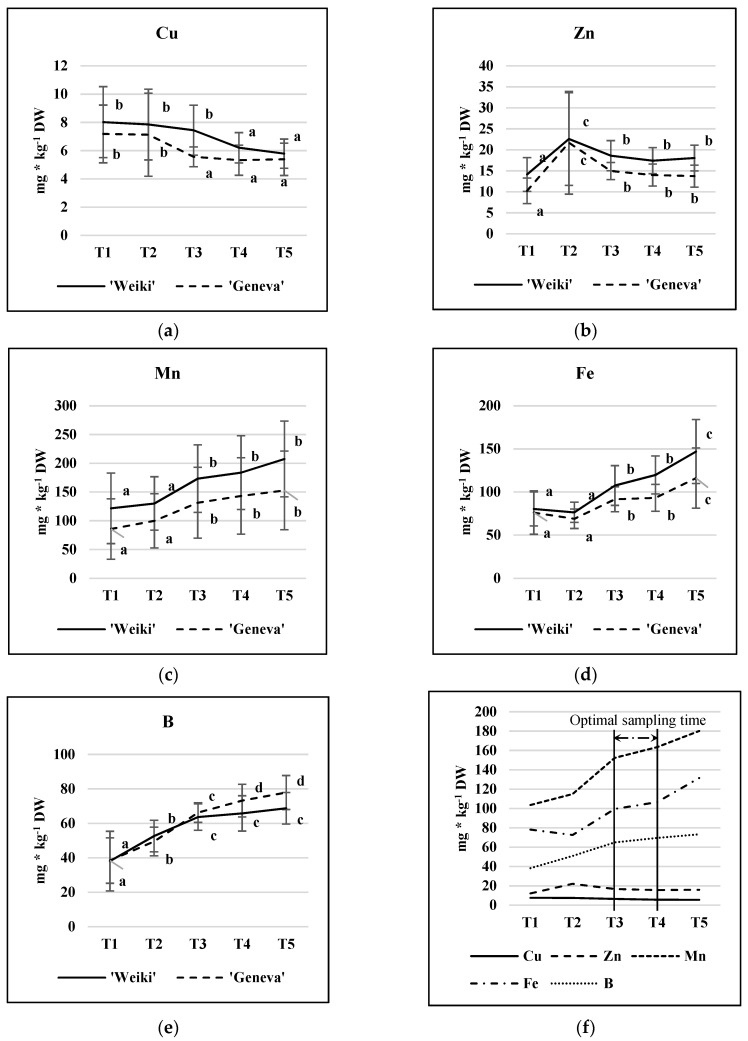
Leaf micronutrient concentration (mg kg^−1^ DW): cultivar and time of sampling effects. (**a**) Cu; (**b**) Zn; (**c**) Mn; (**d**) Fe; (**e**) B; proposed range of sampling time for diagnostic purpose (**f**). Results are averaged over N level treatments and 2015–2016 growing seasons (avg ± SD, n = 18). In each year, leaves were sampled five times (T1—fully developed leaf stage (28.05.15 and 2.06.16), T2—the beginning of intensified fruit growth (23.06 in both, 2015 and 2016); T3—the peak of intensified fruit growth (16.07.15 and 14.07.16); T4—the full development of fruit (06.08.15 and 04.08.16); and T5—the beginning of fruit physiological maturity (27.08.15 and 25.08.16). Values for each cultivar marked with a different letter differ significantly at *p* ≤ 0.05 (Tukey HSD test). Cultivars were processed separately.

The pattern of changes in micronutrient concentration across the vegetation period is presented in Figure 1. As revealed in the statistical analysis, the leaf sampling time significantly affected the micronutrient concentrations (Table 1). The Cu concentration in the leaves of both cultivars decreased during the growing season, with a significant change occurring between T2 and T3 and between T3 and T4 for ‘Geneva’ and ‘Weiki’, respectively (Figure 1a). In contrast to Cu, an increase in concentration was observed for Mn, Fe, and B throughout the season; however, the dynamics of the changes depended significantly on the nutrient (Figure 1c–e). The concentrations of B and Fe increased until the end of the growing season, while the leaf Mn stabilized in the middle of the study period, with slight cultivar differences. Zn concentrations, after a sharp increase at the T2 point, remained stable throughout the season in the leaves of both cultivars (Figure 1b). The increase observed at the T2 point was due to abnormally high readings acquired in 2016 (data not shown). According to the present study, the time period between T3 and T4 (quite stable nutrient levels) could be considered as suitable for leaf sampling for diagnostic purposes regarding all the discussed micronutrients (Figure 1f). This means that the time range of sampling could be between mid-July and the beginning of August in Poland (Central Europe). This is in accordance with the sampling time for leaves for macronutrient analysis as indicated in the previous paper [27]. Moreover, the course of micronutrient changes shown in the Figure 1 are of use with regard to a more precise indication of leaf collecting regarding the nutritional problem concerning an individual micronutrient.

A similar trend in micronutrient leaf concentration over the growing season was found for kiwifruit [21]. Changes in the concentrations of Cu and B in leaves were also similar to another kiwiberry study [34], but the trends for Mn, Fe, and Zn appeared to be the opposite. Moreover, the leaf Mn and B concentrations acquired in that study [34] were generally lower than in the present study, possibly due to differences in soil organic matter, which is important in terms of plant nutrition and micronutrients. In the present study, the soil organic matter concentration was approximately 2% (data not shown), while the kiwiberry plants from the study by Decorte et al. [34] were grown in sand. The B, Fe, Mn, Cu, and Zn concentrations in *A. arguta* ‘Ananasnaya’ found in that study generally expressed the same trend in changes throughout the season as in the present study, but there was also gender effects [30]. The female ‘Ananasnaya’ vines generally had higher concentrations of leaf nutrients than males, particularly in the early to mid-season. In the present study, female plants were examined and, except for B, ‘Weiki’ leaves showed a much higher micronutrient content than ‘Geneva’ (Figure 1), which confirms the influence of genotype on micronutrient uptake efficiency and nutrient demand. The effect of cultivar was also visible in the macronutrient study, where ‘Weiki’ leaves showed higher concentrations of N and P than ‘Geneva’ but lower concentrations of Mg and S [27]. Summing up, the study results revealed that, under the same feeding conditions, different genotypes of the same species can be distinguished in terms of demand, efficiency of nutrient use, uptake, and translocation [14,27,30].

### 2.4. Fruit Micronutrient Concentration: N Level, Cultivar, and Year Effects

*A. arguta* fruit is reported to be a rich nutritional source of mineral elements that are helpful in maintaining human health, e.g., P, K, Ca, Mg, Cu, Zn, and Fe [37,38]. The effect of increasing N fertilization on Cu, Zn, and Fe concentrations in fruits was not proven statistically in the present study (Table 5); however, an increase in the concentrations of those nutrients could still be identified (Table 6). Moreover, based on increasing N nutrition, a significant change in B concentration in ‘Weiki’ fruits was observed, matching the downward change pattern in the leaves of this cultivar. The highest concentration of B in ‘Weiki’ leaves was observed at the lowest (N1) N level, and the lowest B concentration was observed at the N2 level. No change was observed in the B concentration in ‘Geneva’ fruits. In a previous study concerning kiwifruit, an increasing N supply of 30, 60, and 90 kg ha^−1^ resulted in decreased B and Zn concentrations [28]. In the present study, increasing the soil N supply had an unambiguously positive effect on Mn concentrations in fruits, with a significant change between N2 and N3 levels for ‘Weiki’ and between N1 and N3 for ‘Geneva’ fruits. The Mn concentration in kiwiberry leaves followed the same pattern, suggesting an interrelationship between soil N and leaf and fruit nutritional status with respect to this element (Table 4 and Table 6). The positive effect of N supply on Cu, Fe, and Mn, but its negative effect on Zn, were previously documented in studies on corn [39]. No effect in terms of the application of 80 and 160 kg ha^−1^ N on Fe, Mn, and Zn was observed in apple fruits during a recent two-year study, except for a positive change in Zn concentration when between 0 and 80 kg ha^−1^ N was applied [2]. N fertilization of 130 kg ha^−1^ has been reported to increase Fe, Zn, and Cu density in wheat grain, compared with no N fertilization, and to have no effect on Mn concentrations. A further increase in the N dose to 300 kg ha^−1^ did not increase the Fe, Zn, and Cu densities in grain [40]. In contrast to the findings of Shi et al. [40], in the present study, a better N supply positively affected the Mn concentrations in kiwiberry.

There was a contrasting effect in terms of the growing season (‘year effect’) on micronutrient fruit concentrations compared with those observed in leaves. A significant decrease in Zn, Mn, Fe, and B was observed in ‘Weiki’ fruits in 2016 compared with 2015 (Table 7). In the case of ‘Geneva’ fruits, such a decrease was only observed for Zn and Fe. No significant effect in terms of the growing season was noted for Cu in either of the tested cultivars. In a previous study, fruit N, Mg, and Ca concentrations also decreased in 2016 compared with 2015 [27]. The year effect could be related to differences in the temperatures or rainfall amount and distribution between years [2,27].

The average fruit weight in 2016 was significantly higher compared to in 2015, which could have affected the concentration of certain fruit micronutrients (‘dilution effect’). However, no effect in terms of the growing conditions on the leaf blade area was noted (data not shown). These data may partly explain the differences in the year effect on the concentration of micronutrients in fruits and leaves.

## 3. Materials and Methods

The results presented in this paper are a supplement to the previously published study on leaf macronutrient status in relation to an increasing soil N level, cultivar, and sampling time [27], and the experiment design has already been described in detail. Here, therefore, the experimental data are presented as concisely as possible. For a better understanding of the results presented in this manuscript, a short summary of the influence of increasing soil N levels on leaf N content depending on the cultivar in consecutive growing seasons is presented as a background (Table 1).

### 3.1. Plant Material, Weather, and Growth Conditions

Tested samples (soil, leaf, and fruit tissues) were collected in 2015 and 2016 from the commercial *A. arguta* growing orchard (Bodzew, Poland; 51°47′49.9″ N + 20°48′44.0″ E) on a farm that was a partner in the supported project. The female plant tissues were sampled.

Weather data at the experimental site were collected with a field weather station (Vantage Pro 7, Davis Instruments, Hayward, CA, USA). Briefly, compared to the long-term average (1982–2012), the mean monthly temperatures in the examined growing seasons (March–September) were 1.8 and 1.6 °C higher in 2015 and 2016, respectively. In turn, the sums of total precipitation compared to the long-term average were lower by 26% and 6% for 2015 and 2016, respectively. Therefore, 2015 was clearly warmer and drier not only compared to the long-term average but also compared to 2016. More detailed weather data were recently published [36]. Plants of two kiwiberry cultivars, ‘Weiki’ and ‘Geneva’, which are currently grown in several European countries as cultivars recommended for commercial cultivation [29], were selected for the study. The plants were planted in a 4 × 4 m spacing in 2011 and trained on a T-bar support. The total male-to-female ratio on the plantation was 1:7. The experiment was set up in a randomized block design. The experiment consisted of three replications where four female plants per repetition were similarly located in relation to male plants. Soil samples were collected in early to mid-March. The N levels under study were 30, 50, and 80 mg N kg^−1^ dry soil weight (DW) and were marked as N1, N2, and N3 respectively. The N doses for particular N treatments were calculated based on: (i) the available early spring soil N content (the N content in the soil was subtracted from the respective contents/levels under study), (ii) a topsoil depth of 0.2 m, and (iii) a soil density of 1.5 kg dm^3^ on average. Plots were fertilized in three equal doses at the beginning of April, and then four and eight weeks later, using NH_4_NO_3_ containing 34% N. For detailed ranges of the added N as well as other essential nutrients and the pH kept, please refer to the previous paper [27].

Soil macronutrients were extracted using 0.03 M CH_3_COOH, while in the case of micronutrients, the Lindsey solution was used. The nutrient measurements were then conducted according to Komosa et al. [41].

### 3.2. Leaf and Fruit Collection and Analysis

Leaf samples were collected at five time points during the growing season, starting on 28 May in 2015 and on 2 June in 2016 and continuing every three weeks until the end of August, with the time points being marked as T1, T2, T3, T4, and T5, respectively. For each point time, three repetitions per treatment were collected. Each collected repetition consisted of 24 mature leaves, gathered evenly from both sides of every plant from terminating shoots.

Fruit was sampled when their soluble solids concentration (SSC) reached 6.5–7.0%, which is recommended as a suitable time point for kiwiberry harvest [42]. In 2015, fruit was collected on 3 and 9 September for ‘Geneva’ and ‘Weiki’, respectively, and in 2016, on 30 August and 19 September, respectively. Three repetitions for each N treatment were collected, each consisting of ten similar-sized fruits with no visible deformation or damage.

Leaf and fruit samples were oven-dried at 70 °C and ground to a fine powder, with their dry weights being determined after drying at 105 °C. The micronutrient analysis was conducted in the laboratory of the Chemical and Agricultural Station in Warsaw (Poland, accreditation number AB 312, PN-EN ISO/lEC 17025:2005 standard, http://www.oschr-warszawa.pl, accessed on 7 July 2017). Standard procedures were used to measure the total micronutrient concentrations in examined kiwiberry tissues. For the mineralization of fruit and leaf samples, phosphoric acid and hydrogen peroxide were used. Flame atomic absorption spectroscopy (FAAS) at 324.8 nm, 248.3 nm, 279.5 nm, and 213.9 nm was used to determine the Cu, Fe, Mn, and Zn concentrations, respectively. The B concentration was evaluated by inductively coupled plasma optical emission spectrometry (ICP-OES) at 249.678 nm. The results were shown as mg kg^−1^ of dry weight (DW).

### 3.3. Statistical Analysis

The obtained data were elaborated using Statistica version 13.0 software (TIBCO Software Inc., http://statistica.io—access date 7 July 2017, Palo Alto, CA, USA). The significance of the differences between means of main effects was evaluated using Tukey’s test (HSD) at *p* ≤ 0.05. Cultivars were processed separately due to a significant differences in terms of leaf and fruit chemical composition. Leaf analyses included three (year, N-level, time of leaf sampling) main factors to be analyzed, whereas fruit analyses included two (year, N-level). The interactions between main effects were generally not significant, therefore the data were presented as mean values for particular sources of variation, separately for the ‘Geneva’ and ‘Weiki’ cultivars. A summary of statistics data is presented in Table 1 and Table 2.

## 4. Conclusions

The leaf chemical analysis revealed a cultivar-dependent relationship between soil N level and leaf micronutrient concentration. A significant increase in Cu, Zn, Mn, and Fe was noted in ‘Geneva’ leaves when there was a higher N supply, while only the increase in Mn was proven to be significant in ‘Weiki’ leaves. In the case of fruit, increased N fertilization was followed only by a higher Mn content in both tested cultivars. Micronutrient accumulation in *A. arguta* leaves changed significantly during the growing period. Over time, Mn, Fe, and B concentrations increased markedly, Cu decreased, and Zn remained mostly stable. Between mid-July and the beginning of August, the lowest fluctuations in the micronutrient contents were recorded. The effect of the year on leaf micronutrient accumulation was highly significant; except for Fe, significantly higher micronutrient levels were revealed in 2016, which could be considered as hot and wet compared to both the long-term average and 2015 in particular. Compared to the leaves, the growing season effect was much smaller in the case of fruit micronutrient concentrations. Irrespective of cultivar, the N fertilization increase resulted in a higher fruit Mn concentration and was insignificant in the case of other micronutrients. The results indicate that the N dose may affect the uptake and accumulation of micronutrients within a range depending on the tissue type and the genotype. Interrelationships between essential nutrients are one of the most important issues in terms of effectiveness in plant mineral nutrition.

As a short note, we want to add that the results presented in this manuscript can be read with other reports dealing with N effects on the physiology/biochemistry of kiwiberry plants explored under the same conditions as those described in this study [33,36,43].

## Figures and Tables

**Table 1:** Leaf N concentration (% DW) in relation to year, cultivar, and soil N level. Data are means over the season (leaf samples were collected at five time points) ±SD (n = 5).

	Year			
N Level	2015		2016	
	‘Weiki’	‘Geneva’	‘Weiki’	‘Geneva’
N1 *	2.11 ± 0.26 a	1.67 ± 0.22 a	2.35 ± 0.76 a	1.76 ± 0.49 a
N2	2.23 ± 0.24 a	1.69 ± 0.29 a	2.55 ± 0.78 ab	2.23 ± 0.50 b
N3	2.56 ± 0.19 b	1.92 ± 0.31 b	2.65 ± 0.73 b	2.33 ± 0.42 b
Av	2.30	1.76	2.51	2.11

* N1-30, N2-50, N3-80 mg of N kg^−1^ DW. Values in the column marked with a different letter differ significantly at *p* ≤ 0.05 (Tukey HSD test).

**Table 2:** Statistics: values of *p* for particular sources of variation and their interaction depending on the micronutrient and cultivar tested (LEAF tissue).

Cultivar	Nutrient				
‘Weiki’					
*p* value for:	Cu	Zn	Mn	Fe	B
Year (A)	0.0000	0.0000	0.0000	0.0000	0.0000
N level (B)	0.1227	0.1772	0.0000	0.0536	0.0014
Sampling date (C)	0.0000	0.0000	0.0000	0.0000	0.0000
A × B	0.0151	0.5483	0.3827	0.5167	0.3872
A × C	0.0000	0.0000	0.0341	0.0000	0.0000
B × C	0.7412	0.6198	0.2816	0.9676	0.5547
A × B × C	0.5194	0.6136	0.9775	0.9945	0.8882
‘Geneva’					
*p* value for:	Cu	Zn	Mn	Fe	B
Year (A)	0.0000	0.0000	0.0000	0.0001	0.0000
N level (B)	0.0014	0.0032	0.0000	0.0001	0.0496
Sampling date (C)	0.0000	0.0000	0.0000	0.0000	0.0000
A × B	0.0143	0.1387	0.0000	0.7831	0.0756
A × C	0.0000	0.0000	0.5910	0.0000	0.0000
B × C	0.0561	0.1668	0.1515	0.9879	0.2716
A × B × C	0.0401	0.7032	0.9962	0.9271	0.4201

**Table 3:** Leaf micronutrient concentration (mg kg^−1^ DW): cultivar and N effects.

	Cultivar				
	‘Weiki’				
**N Level**	Cu	Zn	Mn	Fe	B
N1 *	6.70 ± 2.10	18.5 ± 5.96	129.3 ± 58.3 a	102.4 ± 33.1	60.7 ± 16.7 b
N2	7.29 ± 2.43	17.5 ± 6.37	148.7 ± 44.2 a	104.6 ± 35.8	54.4 ± 14.8 a
N3	7.19 ± 1.57	18.5 ± 6.76	211.6 ± 67.7 b	111.6 ± 37.3	58.2 ± 15.7 ab
Av^Weiki cv.^	7.06	18.16	163.2	106.2	57.78
	‘Geneva’				
**N level**	Cu	Zn	Mn	Fe	B
N1	5.82 ± 1.99 a	14.0 ± 6.43 a	78.8 ± 23.8 a	80.4 ± 25.3 a	62.0 ± 18.6
N2	6.45 ± 2.04 b	15.1 ± 6.53 ab	121.3 ± 47.3 b	94.3 ± 29.0 b	62.5 ± 19.2
N3	6.08 ± 1.79 ab	15.6 ± 7.88 b	167.5 ± 75.3 c	92.9 ± 25.5 b	58.6 ± 15.2
Av^Geneva cv.^	6.12	14.92	122.5	89.2	61.04

* N1-30, N2-50, N3-80 mg of N kg^−1^ DW. Results are averaged over the season (leaf samples were collected at five time points) and over the growing seasons of 2015–2016 (avg. ± SD, n = 30). Values in the column marked with a different letter differ significantly at *p* ≤ 0.05 (Tukey HSD test). An absence of letters means no N effect on micronutrient content at *p* ≤ 0.05. Cultivars were processed separately.

**Table 4:** Leaf micronutrient concentration (mg kg^−1^ DW): cultivar and year effects.

	Cultivar				
	‘Weiki’				
**Year**	Cu	Zn	Mn	Fe	B
2015	5.81 ± 1.05 a	13.9 ± 2.60 a	138.6 ± 66.1 a	113.7 ± 45.5 b	51.0 ± 18.0 a
2016	8.31 ± 2.07 b	22.4 ± 6.13 b	187.8 ± 58.9 b	98.7 ± 18.4 a	64.5 ± 9.24 b
	‘Geneva’				
**Year**	Cu	Zn	Mn	Fe	B
2015	4.88 ± 0.61 a	10.9 ± 2.34 a	90.9 ± 41.9 a	95.0 ± 36.4 b	57.0 ± 21.0 a
2016	7.35 ± 2.03 b	18.9 ± 7.69 b	154.2 ± 66.8 b	83.4 ± 9.63 a	65.1 ± 12.5 b

In each year, the leaves were sampled five times every three weeks, starting from 28 May in 2015 and from 2 June in 2016. Results are averaged over the season (leaf samples were collected at five time points) and the N1-N3 treatments (avg. ± SD, n = 45). Values in the column marked with a different letter differ significantly at *p* ≤ 0.05 (Tukey HSD test). An absence of letters means no year effect on micronutrient content at *p* ≤ 0.05.

**Table 5:** Statistics: values of *p* for particular sources of variation and their interaction depending on the micronutrient and cultivar tested (FRUIT tissue).

Cultivar	Nutrient				
‘Weiki’					
*p* value for:	Cu	Zn	Mn	Fe	B
Year (A)	0.6341	0.0001	0.0429	0.0040	0.0000
N level (B)	0.1245	0.2159	0.0145	0.1843	0.0187
A × B	0.1690	0.4440	0.7741	0.6727	0.4034
‘Geneva’					
*p* value for:	Cu	Zn	Mn	Fe	B
Year (A)	0.34751	0.00146	0.25462	0.00018	0.08428
N level (B)	0.19829	0.77816	0.03577	0.08700	0.91395
A × B	0.01491	0.06753	0.61358	0.23647	0.03083

**Table 6:** Fruit micronutrient concentration (mg kg^−1^ DW): cultivar and soil N effects.

	Cultivar				
	‘Weiki’				
**N Level**	Cu	Zn	Mn	Fe	B
N1 *	5.91 ± 0.78	9.2 ± 2.97	15.8 ± 6.34 a	31.5 ± 7.47	17.1 ± 2.62 b
N2	6.44 ± 0.96	10.2 ± 3.77	17.1 ± 4.17 a	38.4 ± 9.06	15.6 ± 2.09 a
N3	5.45 ± 0.61	11.7 ± 5.22	25.7 ± 6.45 b	36.0 ± 7.58	16.8 ± 2.22 ab
Av^Weiki cv.^	1.08	0.19	1.24	0.08	0.31
	‘Geneva’				
**N level**	Cu	Zn	Mn	Fe	B
N1	4.69 ± 0.93	7.2 ± 3.54	11.6 ± 4.05 a	23.6 ± 4.44	15.4 ± 1.28
N2	5.20 ± 0.0.58	7.7 ± 2.58	15.6 ± 3.59 ab	23.4 ± 1.78	15.3 ± 0.52
N3	5.39 ± 0.96	7.9 ± 1.29	19.3 ± 5.29 b	26.0 ± 3.77	15.5 ± 1.43
Av^Geneva cv.^	0.62	0.17	1.11	0.08	0.37

* N1-30, N2-50, N3-80 mg of N kg-1 DW. Results are means for 2015–2016 (avg. ± SD, n = 6). Values in the column marked with a different letter differ significantly at *p* ≤ 0.05 (Tukey HSD test). An absence of letters means no N effect on micronutrient content at *p* ≤ 0.05. Cultivars were processed separately.

**Table 7:** Fruit micronutrient concentration (mg kg^−1^ DW): cultivar and growing season effects.

	Cultivar				
	‘Weiki’				
**Year**	Cu	Zn	Mn	Fe	B
2015	6.02 ± 0.83	13.5 ± 3.23 b	22.3 ± 4.88 b	40.5 ± 4.98 b	18.5 ± 1.24 b
2016	5.84 ± 0.92	7.2 ± 1.08 a	16.7 ± 7.96 a	30.1 ± 7.82 a	14.5 ± 0.64 a
	‘Geneva’				
**Year**	Cu	Zn	Mn	Fe	B
2015	5.24 ± 0.55	9.29 ± 2.19 b	14.2 ± 4.00	26.9 ± 2.98 b	15.8 ± 1.02
2016	4.94 ± 1.08	5.98 ± 1.5 a	16.7 ± 6.19	21.8 ± 1.54 a	15.0 ± 1.04

Results are means for the all N treatments (avg. ± SD, n = 9). Values in the column marked with a different letter differ significantly at *p* ≤ 0.05 (Tukey HSD test). An absence of letters means no year effect on micronutrient content at *p* ≤ 0.05.

## Data Availability

Not applicable.
